# Supplementary figures and images for: Use of standard U-bottom and V-bottom well plates to generate neuroepithelial embryoid bodies
Source: PLoS One. 2022 May 10;17(5):e0262062. doi: 10.1371/journal.pone.0262062 (PMC9089918; doi:10.1371/journal.pone.0262062)

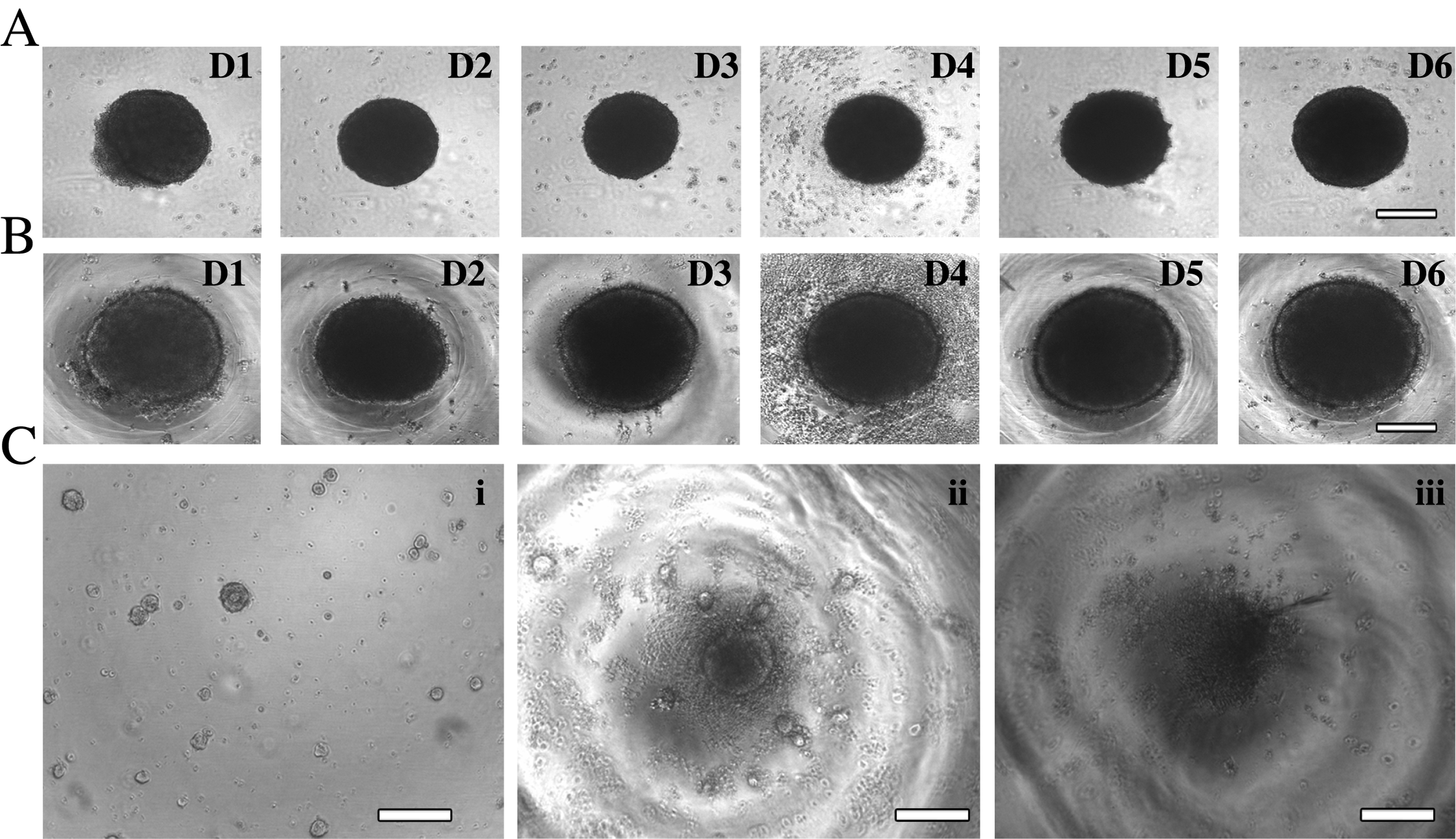

Supplement: S1 Fig — Examples of appropriate EB growth in V-bottom (A) and U-bottom (B) anti-adherence coated plates at different days (“D”). Scale bar: 200 μm. (C) Examples of EBs that fail to consolidate. (i) Low seeding concentrations or non-coated wells produce small cell aggregates. These small aggregates will not fuse together later and will not develop into EBs. Scale bar: 200 μm. (ii) Central cluster with satellite aggregates in non-coated wells. As in (i), these peripheral aggregates will not fuse with the main cluster. Scale bar: 200 μm. (iii) Disintegrated EB; remains of an EB that did not survive until the end of the experiment. Scale bar: 200 μm. (TIF) [file pone.0262062.s001.tif]
