# Supplementary material for: Use of standard U-bottom and V-bottom well plates to generate neuroepithelial embryoid bodies
Source: PLoS One. 2022 May 10;17(5):e0262062. doi: 10.1371/journal.pone.0262062 (PMC9089918; doi:10.1371/journal.pone.0262062)
Supplement: S1 Table — (DOCX) [file pone.0262062.s002.docx]

**Supporting Information**

**Supplementary Table S1. Time and Cost comparison of different embryoid body well-plate formation techniques.**

| Method | Lowest reported cell seeding | Preparation  time | Cost/well plate (USD)* | Reference |
| --- | --- | --- | --- | --- |
| Anti-adherence solution + Centrifugation† | 7,000 | 5 min | low | This work |
| Commercially available ULA plates† | 9,000 | 5 min | high | [10] |
| PolyHema coating | 20,000 | 72 h | medium | [34,35] |
| Agar coating | 10,000 | 30 min | low | [16,36] |
|  |  |  |  |  |
| Polyvinyl alcohol | 5,000 | 6–8 days | low | [21,28] |
| Ad hoc molds |  |  |  |  |
| PDMS (Plasma treatment) | 150,000/mL | 24 h | medium | [12,37] |
| Magnetic molding† | 11,000+ | 1 h | low | [38] |
| Silicone molding | 1,000 | 1 h | Low | [39] |
| 3D printing EBs (gelatin +agar) | 500,000/mL | 30 min | medium | [40] |
|  |  |  |  |  |

† Uses anti-adherence solution.

* Estimated price based on reported materials and manufacturers.
